# Evaluation of dietary habits and cooking confidence using virtual teaching kitchens for perimenopausal women

**DOI:** 10.1186/s12889-023-15509-x

**Published:** 2023-03-31

**Authors:** Sarah Sommer, Andrea Pelletier, Andrea Roche, Laura Klein, Kimberly Dawes, Susan Hellerstein

**Affiliations:** Obstetrics, Gynecology and Reproductive Biology, Harvard Medical School, Brigham and Women’s Hospital, Boston, MA USA

**Keywords:** Teaching kitchens, Perimenopause, Nutrition, Culinary Medicine, Diet, Cooking

## Abstract

**Background:**

The transition to menopause is a time when women are at increased risk for chronic and cardiovascular diseases, and weight gain. This study evaluates the efficacy of virtual teaching kitchen (TK) interventions on cooking confidence and consumption of a healthy diet in women over 45.

**Methods:**

This teaching kitchen intervention is a synchronous online series of classes for perimenopausal women, with 45 min of live cooking and 15 min of nutrition discussion. From September 2020 through January 2022, participants completed online pre- post-intervention surveys addressing weight, eating habits, cooking confidence and self-efficacy. Analysis used paired samples t-test and Wilcoxon signed rank sum test for normally and non-normal distributed data respectively.

**Results:**

Of the 609 unique participants, 269 women completed both pre and post surveys after attending classes. Participants self-reported a statistically significant decreased weight (p < 0.001), increased daily consumption of fruit/vegetables (p < 0.039), fish (p < 0.001) and beans (p < 0.005), and decreased daily consumption of red meat (p < 0.001), sugary beverages (p < 0.029) and white grains (p < 0.039). There was significant improvement in cooking self-efficacy and confidence.

**Conclusions:**

Virtual teaching kitchens were effective in improving culinary and dietary habits among peri- and post-menopausal women. This early evidence suggests that teaching kitchens can effectively reach larger populations for healthy behavioral modification.

**Trial Registration:**

Study obtained IRB exemption.

## Background

Women in the US will spend 30 years, or 40% of their lives in menopause. [1] Although multifactorial in etiology, diet-related chronic diseases like obesity, Type 2 DM, and cardiovascular disease become more prevalent during menopause. Hormonal changes with menopause, particularly lower estrogen levels, increase a woman’s risk for heart disease, diabetes, and changes in body composition. [2, 3] Menopause transition is a time where women’s risk of chronic and cardiovascular disease accelerates and is critical for early interventions to reduce cardiovascular risk and improve eating habits. [4].

The transition to menopause is variable, often beginning between 45 and 55 years old and lasting 7 or more years, with the median age of natural menopause at 51.4 years. According to the 2020 US Census, there are close to 72 million women in the US over 45 [5]. In the US, 42% of women from 40 to 59 years old are obese, and Type 2 diabetes most often develops in people over age 45; the estimated lifetime risk for women born in 2000 of developing diabetes mellitus is 38.5% [6, 7]. The development of heart disease in women increases after menopause such that cardiovascular disease is the number one cause of death and disability. [6] Symptoms of cardiovascular disease in women increases from 11% at age 45–64 to 33% after 65 years old. [6] Coronary heart disease accounts for 22% of all-cause mortality in US women. [8].

Worldwide, poor diet quality was identified as the leading risk factor for high BMI and death from cardiovascular disease and major noncommunicable diseases, [9] and diet has been linked to risk for disease in perimenopause such as breast cancer. [10] The 2020 United States Department of Agriculture (USDA) dietary guidelines, which feature “My Plate,” are aimed to decrease the risk of chronic diseases by focusing on nutrient dense food, calorie guidelines, half of each plate filled with vegetables and fruit, one quarter whole grains, and one quarter lean proteins as well as food and beverage choices with less added sugar, saturated fats, and sodium. However, the diet of most menopausal women in the United States is not aligned with the dietary recommendations; while older adults generally eat more fruits and vegetables and less high-energy foods than younger people, only about half eat the recommended 5 servings of fruits and vegetables per day. [11] Women over age 60 generally consume more than the daily recommended amount of sugar, saturated fat, and sodium, which all contribute to an increased risk of chronic disease. [11] Conversely, fruit and vegetable intake has been associated with a lower risk of mortality and hospitalization in older adults, and a recent review of high-quality diets found an association with lower risk of cardiovascular disease, type 2 diabetes, neurodegenerative diseases and cardiovascular and all-cause mortality. [12].

How can women in the menopause transition and post-menopause effectively be educated about the dietary guidelines and given the skills to implement them? According to a study by Wolfson and Bleich, cooking dinner frequently at home is associated with a healthier diet, whether individuals are trying to lose weight or not. [13] Healthy patterns of cooking and eating can prevent disease and reverse some chronic disease. Cooking skills correlate positively with weekly vegetable consumption and negatively with weekly consumption of unhealthy food groups. [13].

A teaching kitchen (TK) is a physical or virtual learning lab aimed to optimize cooking and eating behaviors through culinary skill building, nutrition education, and recipe strategies. There is evidence that teaching kitchens are valuable tools for behavior change and improvement of eating habits in the workplace [14] and with individuals with type 2 diabetes. [15] They have been demonstrated to significantly change behavior around healthy eating, body weight, waist circumference, blood pressure, and certain biomarkers. [14–16].

Virtual teaching kitchens have become a more widely accepted means of intervention following the COVID-19 pandemic, and our analysis adds to the emerging body of literature about the efficacy of these programs. [14, 17]. The purpose of our program and analysis was to evaluate the efficacy of a virtual teaching kitchen intervention on cooking confidence and the consumption of a healthy diet in women over 45. We hypothesized that for women over 45, using the combined expertise of a doctor, dietitian, health coach, and chef to design and teach virtual cooking classes on Zoom, class participation could improve dietary habits and improve attitudes around cooking confidence and competence.

## Methods

### Study Design

We used pre and post- intervention surveys to evaluate the impact of participation in the NuCook virtual teaching kitchen class educational program. The need for ethical approval and informed consent was waived by the institutional review board of Mass General Brigham(MGB) as this was determined to not meet the criteria for human subject research as defined by Mass General Brigham Human Research policies and Health and Human Services regulations set forth in 45 CFR 46.‘ All methods were carried out in accordance with relevant guidelines and regulations of Mass General Brigham.

### Intervention

Cooking and nutrition classes, co-designed and taught by a physician, dietitian, health coach and chef were offered live over Zoom in a program called NuCook at no cost to participants. From September 2020 through December 2021, 9 courses were offered, with 3–5 virtual classes per course. Participants were given the recipes and instructions in advance to enable a hands-on experience from their own home and could choose to cook in real-time or observe the class. Classes were offered at 6 pm Eastern Standard Time. Participants completed between 1 and 30 classes over 1 or multiple courses.

The class curriculum was developed by updating the in-person curriculum from an initial pilot study, which utilized the educational methodology of Cooking Matters for Adults. [18] The online curriculum used best practices in synchronous, remote education adapting the Culinary Healthcare Education Fundamentals (CHEF) - Coaching Program to direct participant programming, as this was an emerging field given COVID-19. [17] Each class had two healthy recipes, nutrition goals, and culinary goals. Classes and curriculum were improved upon throughout the study period based on participant feedback, such as alterations to class pacing, ingredient substitutions, and increased participant-coach interactions.

The classes focused on a plant-forward approach to healthy eating and eating in accordance with the “Harvard Healthy Eating Plate” which is similar to My Plate but adds guidelines on healthy plant oil consumption. Each class had designated culinary and nutritional teaching goals. Classes had 75 min of instruction, consisting of: 45 min cooking a healthy meal led by a professional chef, 15 min of health and nutrition instruction by a physician and dietitian, and 15 min for questions and community discussion.

### Participant recruitment

Women ages 45 and above with working access to Zoom were eligible to participate in classes taught in English. Recruitment targeted women over age 45 living in East Coast metro areas with interest in cooking, healthy eating, and menopause. Participants were recruited from Mass General Brigham employees through hospital health bulletins, Fish Women’s Health Center patients through EPIC notifications, and from the general population through social media posts on Facebook and Instagram. A NuCook staff member scheduled and sent repeated class reminder emails at regular intervals to all who enrolled including information about recipes and preparation for class. Enrollment was ongoing throughout each course, but capped at 300 participants per class, which was our Zoom capacity. All participants were accepted, but had to complete our enrollment survey to receive the class invitation Zoom link.

### Data collection

Participants were asked to complete a 20-minute pre-intervention survey before beginning the course in order to get a link to the course and a voluntary 20-minute post-intervention survey once they completed each course, administered through Red Cap. Women who participated in more than one course were asked to complete the post-intervention survey again. Survey questions were taken from augmented versions of the National Institute of Health (NIH) ‘Eating at America’s Table Quick Food Scan’ [19] and the ‘Eating Habits Confidence Survey.’ [20] Questions about the Harvard Healthy Plate, [21] whole grain and lean protein consumption, and portion control were added to the validated surveys. Survey questions also included basic demographic information, baseline height and weight and class attendance.

### Data analysis

Participants were assigned a study number linked by the participant’s name and birthdate. Participants’ first pre-survey and last post-survey were used for analysis, regardless of how many courses they completed. Data were analyzed using Stata 16.0. BMI was calculated using subject-reported height and weight. Frequency of food consumed was converted to servings per day. The number of classes taken was self-reported; however, if this data were missing, we used the number of classes recorded by the study staff present at each Zoom session. Total servings of fruits and vegetables were calculated by adding fruit, raw greens, cooked greens, vegetables as protein, and other vegetables. Our analysis used the following definitions: Underweight (BMI < 18.5), Normal Weight (BMI 18.5- <25), Overweight (BMI 25-<30), Obese (BMI > 30).

For continuous, normally distributed data, we reported the mean and standard deviation and used a paired samples t-test to evaluate differences between pre- and post-survey data. For categorical or non-normally distributed data, we reported the using frequency and proportion or median and interquartile range and used the Wilcoxon signed rank sum test to determine differences pre and post. We used multiple linear regression to assess whether the number of classes taken was associated with mean change in outcome variable. Changes pre- and post-intervention were separately analyzed for the group in the lowest quartile for fruit and vegetable consumption at baseline and highest quartile for weight and BMI at baseline using paired samples t-test. Results were considered significant at the p < 0.05. Analysis comparing the lowest quartile for fruit and vegetable consumption habits (< 2.5 servings/day) at baseline to those in the top 75% at baseline was performed. The goal was to capture behavior changes of those with the least healthy dietary habits (lowest fruit and vegetable consumption) at baseline.

## Results

A total of 609 unique individuals were enrolled in the program across 9 cohorts; 44% (N = 269) completed the requirements for analysis (completed pre- and post- course surveys and attended at least one live virtual class). Mean number of classes per participant was 8.2 classes.

The mean age of NuCook participants was 58.8 years old. Of 210 participants surveyed on race and ethnicity, 88.4% were White (n = 183), 6.7% were Hispanic (n = 14), 4.3% were Asian (n = 9) and 1.4% were Black (n = 3).

Mean self-reported weight at baseline across cohorts was 167.6 pounds and mean BMI at baseline was 28.7. At baseline 0.7% were underweight, 37.4% were normal weight, 32.2% were overweight, 29.7% were obese.

Of the 609 unique participants who completed a pre-survey and attended at least 1 class, 269 (44%) completed the post-survey. We compared the 340 individuals who did not complete the post-survey with the 269 study participants. While there were no differences in baseline weight between the groups, there were some differences in dietary habits with those completing the post-surveys consuming higher servings of vegetables as protein, fruit, cooked greens, other vegetables, and beans at baseline and in the response that “cooking takes too much time” (all p < 0.05).

Participants who completed pre- and post-surveys (N = 269) self-reported on average a small but statistically significant weight loss. There was a decrease in self-reported weight from 167.6 pounds on average at baseline to 165.7 pounds after the intervention (p < 0.001). Further analysis by baseline BMI showed weight loss for those self-reported overweight (161.5 to 159.6, p = 0.028) or obese (219.8 to 215.5, p = 0.030) at baseline but not for the normal weight group (135.6 to 136.0, p = 0.514). There were too few subjects in the underweight group to assess changes (n = 2). There was no significant decrease in BMI overall. There was a significant decrease in mean BMI after the intervention for participants who were obese at baseline, (36.7 to 36.0, p = 0.026). Change in weight and BMI was not observed for those at baseline normal or underweight.

Program participants had lower BMI at baseline than the general US female population with a mean weight of 167.6 lbs compared to the average US woman 178.1 lbs (ages 40–49) and 173.5 lbs (ages 50–59).(17) Additionally 32.2% of program participants were overweight and 29.7% obese, compared to the general female USA population where 27.5% are overweight and 41.9% are obese. [22].

Average consumption of fruits and vegetables increased significantly from 3.7 servings per day to 3.9 servings per day (p < 0.039). Participants consumed less servings of red meat (p < 0.001) and more fish (p < 0.001) and beans (p < 0.005). Participants also reported a decrease in less-healthy eating behaviors, including a decrease in sugary beverage consumption (p < 0.029) and intake of white grains (p < 0.031). [Table 1]

**Table 1:** Reported Pre to Post-Intervention Changes in Weight Status and Dietary Habits (n = 269)*

	Pre-intervention	Post-intervention	p value
Weight status			
Weight in pounds, (mean, SD) n = 255	167.6 (2.8)	165.7 (2.7)	< 0.001
BMI (mean, SD) n = 179	28.7 (0.6)	28.5 (0.5)	0.171
**Change in Reported BMI (n, %)**			
Underweight (BMI < 18.5)	n/a	n/a	n/a
Normal Weight (BMI 18.5- <25)	22.7 (0.2)	22.9 (0.2)	0.231
Overweight (BMI 25-<30)	27.6 (0.2)	27.3 (0.3)	0.062
Obese (BMI > 30)	36.7 (1.0)	36.0 (1.0)	0.026
**Dietary habits frequency (Servings/day)**			
Frequency of processed meats (mean, SD)	0.17 (0.02)	0.14 (0.02)	0.137
Frequency red meat (mean, SD)	0.21 (0.02)	0.16 (0.01)	< 0.001
Fish (mean, SD)	0.19 (001)	0.24 (0.01)	< 0.001
Vegetables as protein (mean, SD)	0.76 (06)	0.72 (0.04)	0.454
Whole grains (mean, SD)	0.51 (0.04)	0.56 (0.03)	0.155
White grains (mean, SD)	0.30 (0.03)	0.25 (0.03)	0.031
Fruit (mean, SD)	1.4 (0.06)	1.5 (0.06)	0.425
Raw greens (mean, SD)	0.80 (0.04)	0.89 (0.04)	0.017
Cooked greens (mean, SD)	0.34 (0.03)	0.38 (0.02)	0.132
Other vegetables (mean, SD)	1.10 (0.05)	1.16 (0.05)	0.236
Fruits and vegetables total	3.67 (0.14)	3.90 (0.13)	0.039
Beans (mean, SD)	0.26 (0.02)	0.32 (0.02)	0.005
Sugar beverages (mean, SD)	0.18 (0.02)	0.13 (0.02)	0.029
Fruit juice (mean, SD)	0.11 (0.02)	0.09 (0.01)	0.157

Sense of self-efficacy in healthy eating and living habits also improved across multiple measures. Participants reported feeling more confident that they could follow the balanced plate model on a daily basis (p < 0.011) and minimize added salt by using spice and other flavors (p < 0.017). [Table 2] Women also reported improvements in cooking confidence for the following questions: cooking at home was not too time consuming (p < 0.001), cooking is not frustrating (p < 0.014), and cooking is not too much work (p < 0.001).

We analyzed the mean change in eating behaviors of vegetable and fruit consumption for those participants in the lowest quartile (< 2.5 servings /day) of fruit and vegetable consumption baseline compared to those in the top 75% at baseline [Table 3]. Those at the lowest quartile of fruit and vegetable consumption at baseline had the largest improvements in vegetable consumption in 3 measures: consumption of raw greens (p < 0.018), consumption of vegetables as a source of protein (p < 0.017), and consumption of other vegetables (p < 0.003) compared to those in the top 75%. There was a non-statistically significant trend of increased fruit and cooked greens consumption and improved cooking skills for those in the lowest quartile at baseline. [Table 3]

**Table 2:** Pre- to Post-Intervention Changes in Eating and Cooking Confidence (Likert Scale 1-5) n=269

How sure are you that you can: 1 =“I know I cannot”- 3= “Maybe I can”, 5 =“I know I can”	Pre-intervention Mean	Post-intervention Mean	p value*
Follow the **balanced plate** model on a daily basis?	4.1 (0.1)	4.2 (0.1)	0.011
Eat **whole grains** instead of refined grains?	4.6 (0.1)	4.5 (0.1)	0.896
Eat at least 4–5 servings **fruits & vegetables** a day?	4.3 (0.1)	4.3 (0.1)	0.946
Consume foods or beverages with **less added sugar**?	4.7 (0.05)	4.7 (0.05)	0.903
Choose **lean meats** (chicken, turkey, fish, plant based) instead of red meat?	4.6 (0.05)	4.7 (0.05)	0.156
Use of spices & flavors in cooking to **avoid adding salt** at the table?	4.4 (0.1)	4.6 (0.05)	0.017
Can avoid eating **portions** that are too large?	3.9 (0.1)	4.0 (0.1)	0.128
**Stay physically active** during life changes or when you have excessive demands at home, work, etc.	3.9 (0.1)	4.0 (0.1)	0.035
Can **devote time for physical activity** for at least 20 min most days of the week/5 times per week?	4.1 (0.1)	4.1 (0.1)	0.893
**Views about cooking (Likert 1–5)** (1 = Strongly Disagree, 2 = Disagree, 3 = Neither Agree nor Disagree, 4 = Agree, 5 = Strongly Agree)			
Cooking takes too much time. (mean, SD)	2.8 (0.1)	2.6 (0.1)	< 0.001
Cooking is frustrating. (mean, SD)	2.3 (0.1)	2.2 (0.1)	0.014
It is too much work to cook. (mean, SD)	2.6 (0.1)	2.3 (0.1)	< 0.001
My cooking skills are: (1 = poor, 2 = fair, 3 = good, 4 = excellent) (mean, SD)	3.0 (0.04)	3.0 (0.03)	0.190

**Table 3:** Changes in Weight Status and Dietary Habits by Baseline Quartile of Fruit and Vegetable Consumption

	Mean change Lowest quartile	Mean change top 75th percentile	p value*
Weight	-1.06 (0.65)	-2.11 (1.03)	0.440
Total weekly restaurant meals	-0.18 (0.25)	-0.10 (0.12)	0.757
**FREQUENCY (Scale 0–10)**			
Frequency processed meats	-0.15	-0.17	0.909
Frequency red meat	0.40	0.20	0.098
Fish as protein	0.33	0.20	0.287
Vegetables as protein	0.57	-0.09	0.017
Whole grains	0.28	0.27	0.957
White grains	-0.32	-0.22	0.620
Fruit	0.33	-0.02	0.087
Raw greens	0.65	0.18	0.018
Cooked greens	0.47	0.13	0.086
Other vegetables	0.67	0.01	0.003
Beans	0.43	0.16	0.072
Sugar beverages	-0.38	-0.28	0.605
Fruit juice	-0.17	-0.08	0.521

Analysis of the number of classes attended by each participant was not significantly associated with change in weight, eating habits, or cooking confidence. Lastly, high satisfaction with courses was found, with 95% of participants choosing to continue receiving communications following their first course.

## Discussion

This pre- and post-intervention evaluation showed that the NuCook virtual nutrition and cooking education program significantly decreased weight and improved dietary habits and cooking confidence for the peri/postmenopausal women participants. The program’s effect on weight loss was greatest for those who were overweight or obese at baseline and the improved dietary habits most for those with the least healthy baseline habits.

Since a majority of those who participated did not answer the post survey even though they may have continued to come to class we analyzed their baseline characteristics compared to those who completed the post survey. There was not a difference in age or baseline weight/BMI in the two groups. Evaluation of the differences between those who completed the post-surveys and those who did not, found that those who completed the post class survey at baseline had higher self-reported consumption of fruit, vegetables, vegetable proteins and fewer responded that cooking took too much time. This suggests that those who completed the post survey may be more “health-oriented.” This may add some selection bias in the analysis of those who completed the survey. There may be some participant bias with responses aimed to please us as teachers and researchers. Thus we may not be able to generalize our findings to a broader, less health oriented population. However, we did not have a way of differentiating those who completed the courses but failed to complete a post-course survey from those who dropped out of the classes early. Therefore, further research should be completed to assess whether our results showed response bias or reflected the course content being too advanced or unappealing for individuals less “health-oriented.” Additionally completion of the post-survey was voluntary and we did not have a way of requiring completion for class participation and in future courses we may introduce incentives to reduce this possible study design bias.

Consistent with other published papers on teaching kitchen efficacy, our program participation improved self-reported dietary habits such as the number daily servings of fruits and vegetables, higher fish and bean consumption and a decrease in red meat, white grains, and sugary beverages. [23, 24] Our results regarding weight and BMI were consistent with the aim of the program, which was an approach to healthy cooking and eating based on an understanding of evidence-based culinary and nutritional concepts. At the time of this study, there was little information regarding best practices for teaching kitchens or evidence that teaching kitchen programs could be translated successfully to a virtual format. This analysis added to the growing body of teaching kitchen best practices, while extrapolating techniques to the perimenopausal population. Perimenopause is understudied relative to other phases in the reproductive lifespan; our analysis highlights the importance of interventions that target this population directly.

The menopause transition, with bothersome symptoms, predictable weight gain, and a change in life phase, can be a window of opportunity for behavior change. The psychological literature suggests generally that life transitions can be an opportunity to promote sustainable shifts in habits. [25] Our analysis suggests that perimenopause should be treated as a life transition, with interventions that target this time period. Community and social support are also documented to assist in weight loss and maintenance. [26] The effect of our ability to create community among participants through interactions and shared experiences may have contributed to the program’s success. Further analysis should be performed to assess the role community structure played in perimenopausal participants’ results.

This analysis builds upon and adds to the growing research showing the efficacy of online teaching kitchens to improve cooking confidence, eating habits, and health. The format of the sessions combined hands-on virtual cooking at home with a short didactic nutrition lesson and an opportunity for open discussion. Studies in the education world show that active learning, such as the use of teaching kitchens, is more effective than traditional lectures. [27] A study of 75 veterans within the Overton Brooks Veterans Affairs Medical Center who completed 12-week cooking and nutrition courses either virtually or in-person found significant decreases in weight and improvement of cooking confidence, with no significant difference between those who completed in-person versus online classes. [24] Our high class attendance and retention may show that active-learning oriented, online classes benefit adults by being more easily accessible than in-person counterparts.

Our overall population had lower BMIs and eating habits that were better aligned with dietary recommendations at baseline than the general US population. At baseline, participants were consuming 3.7 portions of fruits and vegetables /day compared to the US average of 2.7 portions/day and drinking 0.1 portions/day sugary beverages compared to 2.6 portions/day.(19) At completion of the program, participants consumed an average of 3.9 servings per day of fruits and vegetables, an improvement from baseline but still under the USDA guidelines recommending 5 servings of fruits and vegetables per day. Given this data, was our program just reaching the healthy-well participants? To assess, we analyzed those participants who carry the highest burden for chronic disease, [28] the at-risk participants with the highest BMIs and lowest baseline fruit and vegetable at baseline compared to the rest of the participant population. We found this culinary medicine intervention was most effective in those at-risk participants with self-reported weight loss for those overweight or obese at baseline but not for the normal weight group and with the largest improvements in vegetable consumption for those at lowest baseline consumption. Further analysis is needed to understand the generalizability of these findings given that the baseline habits were healthier and BMI of our participant population was lower than the general US population.

Population surveys show that 72% of Americans are confused by the conflicting dietary information in the media. [29] As nutrition, wellness, and cooking social media accounts have become more common, misinformation has proliferated. The NuCook curriculum created by a team of professionals including a chef, dietitian, doctor, and health coach, distilled the culinary and nutritional information into concise, clear, and evidence-based messaging. Our virtual format allowed individuals to access professionals in their own homes, where teaching could be directly put into action.

There are some limitations to our findings. Our study population was predominantly white women who, on average, reported healthier baseline dietary habits and lower weights and BMIs than the average US population of perimenopausal women. The homogeneity of our study population yielded inadequate statistical power to assess how age, race, and other demographic characteristics may affect program outcomes, and limits the generalizability of our results. The self-selected participants may be more health-oriented than the average menopausal woman and therefore more susceptible to positive behavior changes. Our sample size is small and needs further validation in a larger population.

The pre- and post- course surveys were self-reported, which may limit the accuracy of the data. The participants self-reported food frequency but not portion size on the food frequency questions. They were given instructions about an average serving size (i.e. 1 cup of raw leafy greens, ½ cup cooked) and asked to report frequency. An initial pilot study of 30 people found that asking about portion size on the self-reported survey led to highly inaccurate results and thus was removed from the survey for this study. However, without information about portion sizes, the portions being eaten may be highly variable. There may also be a social desirability bias for our participants to report to the class teachers. In analyzing participant data, we found that there was not an observable dose-response relationship between the number of classes attended and mean change in behavior and eating habits. This was unanticipated and may reflect our inaccuracies in capturing the number of classes attended in real time or by watching class recordings.

The increasing prevalence of obesity and other diet-related diseases compels educators to develop creative interventions to improve dietary practices and nutritional health outcomes. Virtual teaching kitchens bring valuable culinary and nutrition information into the kitchens of women over age 45, where they can apply what they learn into practice with short-term behavior change that may lead to long-lasting habits.

## Data Availability

The datasets used and/or analyzed during the current study are available from the corresponding author on reasonable request.

## Declarations

No individual personal data is used in any form.

## List of Abbreviations

TK: teaching kitchens
MGB: Mass General Brigham
